# Boy/Girl Friend and Virginity Values, and Stigma Related to Condom Among Jimma University Students

**DOI:** 10.4314/ejhs.v20i3.69446

**Published:** 2010-11

**Authors:** Fentie Ambaw, Andualem Mossie, Teshome Gobena

**Affiliations:** 1 Department of Health education and behavioral sciences, Jimma University; 2 Department of Physiology, Jimma University

**Keywords:** virginity value, boy/ girl friend value, stigma to condom

## Abstract

**Background:**

Individual factors such as the value given to virginity and boy/ girl friend, and stigma attached to condom can strongly affect success in preventing early sexual initiation and in using condom consistently. To the best knowledge of the authors, no literature was available in Ethiopia on these issues until the time of this study. The objective of this study was to assess the value given to virginity, boy/ girl friend, and stigma related to condom access and use.

**Methods:**

A cross-sectional survey was conducted on a random sample of 1986 students in May 2009 in Jimma University using both qualitative and quantitative techniques. Data were collected using piloted and pre-coded questionnaire and from six focus group discussions. Quantitative data were analyzed using SPSS for windows version 13 where descriptive statistics, ANOVA, and t-test were computed. P-value less than 0.05 was considered statistically significant and effect size was measured in Eta squared. The qualitative data findings were triangulated with the quantitative ones.

**Results:**

Of 1986 respondents, 1612 (81.2%) were males, 365 (18.4%) females and 9 (0.4%) their gender not indicated. The age of respondents ranged from 17– 45 years with median of 20 years. Virginity value-scores were significantly lower among females (p< 0.001, Eta squared= 0.023). In contrast to many males, most females appeared to be not concerned about virginity during the focus group discussions. Many respondents of both genders reported that boy/girl friend is very important in campus life. Although the stigma to condom was slightly higher among females (p< 0.001, Eta squared= 0.009), most respondents of both genders had a stigmatizing attitude.

**Conclusion:**

Lower virginity value among females with high value given to boy/girl friend appeared to indicate the liberalization of sex. Liberalization of sex and stigma to condom were occurring together. Sex educators targeting university students should focus on problems of liberalized unprotected sex in relation to success in life.

## Introduction

Virginity is the practice of voluntarily refraining from some or all aspects of sexual activity for reasons such as chastity, prevention of unwanted pregnancy and sexually transmitted infections (STIs), or psycho-sociological reasons such as negative past experiences (1).

Early initiation of sexual activity is linked to higher numbers of non-marital sex partners, minimal condom use, increased rates of STIs, increased rates of out-of-wedlock pregnancy and birth, increased single parenthood, decreased marital stability, increased maternal and child poverty, increased abortion, increased depression, and reduced happiness (2–6).

These consequences incur a high cost to society (7).

The available literatures in the area strongly recommend that sexual behavior among in-school youth and adolescents should be based on the ability to delay sexual initiation and to use condom consistently when abstinence is not possible. Success in these two strategies can be strongly affected by individual factors such as the value given to virginity and boy/ girlfriend, and stigma attached to condom (2, 3). However, to the best knowledge of the authors, no literature was found in Ethiopia on the value given to virginity, boy/ girl friend and stigma to condom up until this study has been undertaken.

The purpose of this study was to address the aforementioned gap in the area. Findings of this study would enable reproductive health educators to select contents of sex education that do not conflict with values of their target , and guide health educators if the need arises to develop values that enhance the overall development of sexual values among in-school adolescents and youth.

## Subjects and Methods

This cross-sectional survey was conducted in Jimma University, main campus in May 2009 using both qualitative and quantitative techniques on 1986 randomly selected regular undergraduate students. The same study subjects as Ambaw et al. (8) were used in this study. To determine the sample size, a single population proportion formulae was used taking 95% confidence interval and 3% margin of error. The variable considered for sample size determination was the estimated prevalence of sexual intercourse among the source population, 32%. This variable was selected because it maximized the sample size. A contingency of 10% and a design effect of 2 were used. This resulted in a total sample size of 2044. Single stage cluster sampling technique was used to select 31 lecture classes of students using lottery method among a total of 206 clusters and all the members in the selected clusters were considered for the study. The qualitative part of the study included six focus groups of 7–10 purposively selected self-expressive participants who were not included in the quantitative section. Students from both gender and year level were included.

The dependent variables of the study were virginity value, boy/ girl friend value, and stigma related to condom. The dependent variables were measured using five-point Likert scale developed by the investigators. There were 5 items in the virginity value scale, 6 items in the boy/ girl friend value scale, and 4 items in the stigma related to condom acess and use scale. The items were totaled in each of the scales to get an interval level of measurement as recommended by Streiner and Norman for data that is not severely skewed (9). Normality of distribution was checked by observing expected normal probability plots rather than histograms and Kolmogorov-Smirnov tests as recommended by Tabachnick and Fidell for large sample size (10).

Professionals in the field of sociology and psychology were consulted to assure content validity. Internal consistency of the scales was checked using Cronbach's alpha test after administering on a randomly selected class of 40 students before the actual study (these were later excluded from the main study). On the 40 sample of students the virginity value scale had a reliability of 0.80, the boy/ girlfriend value scale had a reliability of 0.83, and the stigma related to condom scale had a reliability of 0.81.

Independent variables of the study included socio-demographic characteristics such as age, sex, ethnicity, religion, and church/mosque attending habit, education year level, faculty, and place of origin (rural or urban). Independent variables were measured using self reports on a questionnaire developed by the investigators.

The quantitative data were collected by trained Jimma University instructors using a self-administered, piloted, and precoded questionnaire prepared in simple English in classrooms arranged for that purpose. Supervision was made to assure independence of responses and the questionnaires were collected back immediately after completion by the respondents.

The qualitative data were collected by two trained and experienced facilitators in each focus group who used tape recorders and took notes to document the discussion. Each focus group took 40–75 minutes depending on the range of ideas generated by the study participants.

Guiding questions used for the focus group discussion were: Is virginity important? How? Is boy/ girlfriend important in a campus life? How? What do you feel if you see a student in your campus carrying condom or if you are seen by others carrying condom?

The quantitative data were analyzed using SPSS for windows version 13.0. The independent-samples t-test, one-way between groups ANOVA with post hoc Tukey tests, and descriptive statistics were computed. Normality of distribution and equality of variance were checked before t-test and ANOVA tests were computed. Effect sizes of t-test and ANOVA tests were measured in Eta squared values. A p-value of less than 0.05 was taken as statistically significant. The data from the qualitative part of the study was transcribed, coded and categorized, and finally triangulated with the quantitative findings.

The proposal was reviewed and ethically cleared by Jimma University and informed consent was obtained from every one of the participants before data collection.

The following operational definitions were used:

- **Virginity**: A condition of remaining totally abstinent from sexual intercourse (penis to vagina sex).
- **Virginity value**: Belief about virginity that serves as a guideline to help individuals make decisions whether to remain virgin or to get a virgin partner. High score on the virginity value scale shows high value to virginity and vice versa.
- **Boy/ girlfriend**: Opposite sex friend with whom one has sexual relationship.
- **Boy/girlfriend value**: A belief about boy/girl friend that serve as a guideline to help individuals make decisions about whether to have a boy/girl friend or not. High score on the boy/girl friend value scale shows high value given to boy/ girl fiend in campus life and vice versa.
- **Stigma related to condom:** A negative attitude towards people carrying condom with them. High score on the condom stigma scale shows high stigma to condom and vice versa.

## Results

The detail background characteristics of study participants had been reported in another article (8). To give a brief summary, out of 1986 respondents, 1612 (81.2%) were males, 365(18.4%) females and 9 (0.4%) with their gender not indicated. One thousand seven hundred and thirty one (88%) were single, and 1082 (54.6%) were year-one students in the University. They were also in the age range of 17–45 years (median =20). One thousand forty six (53.4%) were Orthodox Christians, 832 (42.8%) were Oromos, and 1016 (51.8%) had rural origin.

Virginity value had normal distribution with a median value of 19 with scores ranging from 5–25. The independent samples t-test analysis showed that the mean virginity value-scores were significantly higher among males (p< 0.001, Eta squared= 0.023), those who had the habit of frequently attending church/mosque (p< 0.001, Eta squared= 0.017), and those who had rural origin (p< 0.001, Eta squared= 0.029) compared to their counter parts.

Findings from the qualitative part of the study were also more or less similar. For example a fourth year male student made the following remark while expressing the concern he has for the virginity of his anticipated partner:

A girl with previous sexual experiences undoubtedly has different memories of different people. I may not be as good as her previous mates. Even she may think of those people- even when I kiss her. This is a risk no one wants to take, but these days we do not have other options. Really those who are being rewarded for their virginity deserve the rewards.

Another third year male student added:

If a girl is not virgin when you get her, how can you be sure that she is not continuing with her previous relationships! It is very difficult to trust her.

Some male participants were observed even making over generalizations such as “everybody values virginity.” A third year male student, for example, said:

Every body values virginity. But once you are in the extremely strong sexual feeling, just you do it out of your value. (Long laughter exploded).

Many male participants of rural origin had the stand that virginity is important. A third year male student reported:

I have many friends who had not practiced sex. Clever students from rural areas are virgin. We believe that sexual exposure weakens academic performance and it is not acceptable religion wise.

Some male participants considered the value of virginity as if it is on its last legs. A fourth year male student expressed it as:

Culturally virginity was seen as something very dignified. Now a day, that is not the case. Premarital sex is absolutely necessary to rule out sexual incompatibility which is a very important cause of divorce. In this university, many boys and girls do not care about virginity. Girls prefer experienced boys. Because a lot is being said about impotency these days, males also prefer experienced girls to avoid doubts about their ability.

On the other hand the majority of female participants had the opinion that remaining virgin until marriage is not an issue to bother about. For example a second year female student expressed her view as:

Previously it was believed that a girl loves who disvirgined her more than any other male. Now our observations show us that it is not necessarily true. Many are loosing their virginity just casually. Males also know what type of sexual life they have. So, for me, there is no need to worry about my virginity.

Another second year female student added:

You know, in the past if a girl is a virgin, her dignity increases and that makes her preferred for marriage. Now I am educated. The man who comes to me for marriage should come because he loves me not because I am a virgin. Another point is that males do not believe a girl if she tells them that she is virgin unless they check it out themselves. A friend of mine told a boy that she was a virgin, but he replied, “If that is so, my mother is also a virgin.” In such circumstances, how can a girl get the pride of virginity by staying virgin!

Regarding the virginity of anticipated male partner, almost all female participants had expressed its merit. However, female participants seemed to ignore it for the absence of a method to check male virginity, and the females' belief that every male is sexually experienced. For example, a first year female student described it as:

I do not ask a boy “have you ever had sex” because I am 99% sure that he had already done it.

A second year female student added,

If there is a boy with no sexual experience, it is not because he needs it but it is because he is afraid of going about it. (Laughing exploded).

The one-way between groups ANOVA results showed that level of virginity value had a statistically significant difference among respondents of different ethnicities (p< 0.001, Eta squared= 0.019), faculties (p= 0.003, Eta squared= 0.01), age groups (p< 0.001, Eta squared= 0.008), and religions (p< 0.001, Eta squared= 0.018). Post hoc comparisons using Tukey test showed that the mean virginity value score among Oromos was significantly higher than Amharas, and the mean score of Amharas was in turn significantly higher than Gurages. Students of public and medical faculties had significantly higher mean virginity value scores compared to students of humanities and social sciences faculty. Students in the age group 20–24 years had higher mean virginity value score than in the age group 17–19 years. Likewise, Muslims and Protestants had higher mean virginity value scores compared to Orthodox Christians. All other differences observed in the one-way between-groups analysis test were not statistically significant (table 1).

**Table 1:** Comparison of the mean scores of virginity value among the different groups of Jimma University students using One- way between-groups ANOVA and Independent-samples T-test, May, 2009.

Characteristics		Number	Mean	T/F	DF	p-value	Eta squared
**Gender**							
	Male	1604	19.17	6.744	1966	<0.001	0.023
	Female	364	17.47				
**Church/mosque attending habit**							
	Yes	1713	19.07	5.764	1951	<0.001	0.017
	No	240	17.34				
Place of origin*							
	Urban	940	18.11	−7.559	1911.46	<0.001	0.029
	Rural	1012	19.6				
**Ethnicity**							
	Amhara	616	18.62	7.275	(5, 1930)	<0.001	0.019
	Oromo	830	19.38				
	Tigre	57	18.89				
	Gurage	135	17.08				
	Wolita	51	18.61				
	Others§	247	19.02				
**Faculty**							
	Education	108	19.31	3.274	(6, 1969)	0.003	0.010
	Public	429	19.26				
	Business and economics	149	18.27				
	Technology	97	18.16				
	Humanities and social sciences	417	18.31				
	Law	182	18.63				
	Medical sciences	594	19.17				
**Age**							
	17–19 years	420	18.13	8.159	(2, 1938)	<0.001	0.008
	20– 24 years	1435	19.08				
	25–45 years	83	18.42				
**Religion**							
	Orthodox	1042	18.30	11.944	(3, 1946)	<0.001	0.018
	Muslim	344	19.32				
	Protestant	479	19.63				
	OthersΨ	85	19.09				

* equal variance not assumed (p- value of Levene's test =0 .014),

§ include Sidama, Keffa, Silte, Agew, Gamo, Somale, Shinasha, Yem, Dawro, Hamer, hadya,

Ψ Others**= include catholic, “waqofetta”, no religion

Boy/girlfriend value had a normal distribution with median of 14 with scores ranging from 6–30. The independent- samples t-test analysis showed that boy/ girl friend value was higher among males (p= 0.002, Eta squared= 0.005), those who did not have the habit of frequently attending church/ mosque (p< 0.001, Eta squared= 0.012), and those who had urban origin (p< 0.001, Eta squared= 0.013) than their counter parts.

In the qualitative part of the study, the effect of gender on boy/girl friend value was not apparent. Rather two clear categories emerged from both genders standing against each other: The first category is the one that gave high value to boy/ girl friend and emphasizing its being source of pride, way of getting someone to share idea with, an indicator that a girl is not promiscuous, and winning respect from others (including dorm mates). A third year male student for example described the pride of having a girl friend as:

If you are seen with a girl even for a few days, it is a huge pride. Others who want to have a girl friend consult you; no one criticizes you for simply following after the tail of girls…yes, it is a pride!

A second year female participant has also added:

You see I can tell my boy friend what ever bothers me. No one can listen to me as attentively as he does and no one can help me with all what he has as he does.

The second category is the category that emphasized the disadvantages of a boy/ girl friend including wasting time, broken heart as a result of broken relationships (mainly the males were reported to break the relationship), loss of freedom (the boy/ girl fiend has to permit to go to parties, even to go outside the campus), and decreased social interaction with other students. A fourth year male student described the likelihood of breaking a relationship in the university because of the high possibility of getting new sexual partners as:

Seblewongel and Bezabih in the book “Love unto Crypt” (“Fikir Eske Mekabir,” the title of a well known Amharic Novel written by Hadis Alemayehu, Ethiopia) loved each other until their death because they had no choices. But in this university, there are too many Bezabihs for Seblewongel and too many Seblewongels for Bezabih. (Many smiles and noddings).

Results from the one-way ANOVA also showed that level of boy/ girl friend value had a statistically significant difference among groups of different educational year levels (p< 0 .001, Eta squared= 0.018), religions (p< 0.001, Eta squared= 0.013), and age groups (p= 0.002, Eta squared= 0.007). Post hoc analysis using Tukey test showed that the mean score of boy/ girl friend value was significantly lower among students in the age group 17–19 years compared to 20– 24 years or 25– 45 years. But there was no statistically significant difference between age groups 20– 24 years and 25– 45 years. Orthodox Christians and Protestants had significantly higher boy/ girl friend values compared to Muslims, and year-three and above students had higher boy/ girl friend value compared to year-one and year-two students. But there was no statistically significant difference between year-one and year-two or year-three and year-four and above. Boy/ girl friend value scores did not show statistically significant difference among the faculties (table 2).

**Table 2:** Comparison of the mean scores of boy/girl friend value among the different groups of Jimma University students using One- way between-groups ANOVA and Independent-samples T-test, May, 2009.

Characteristics		Number	Mean	T/F	DF	P-value	Eta squared
Gender*							
	Male	1604	15.02	3.163	574.579	0.002	0.005
	Female	364	14.12				
**Church/mosque attending habit**							
	Yes	1713	14.63	−4.861	1951	<0.001	0.012
	No	240	16.34				
**Place of origin**							
	Urban	940	15.47	5.07	1950	<0.001	0.013
	Rural	1012	14.28				
**Level of education**							
	Year-one	1075	14.3	11.969	(3, 1969)	<0.001	0.018
	Year-two	313	14.9				
	Year-three	298	15.6				
	Year-four or above	287	16.1				
**Religion**							
	Orthodox	1042	15.25	8.535	(3, 1946)	<0.001	0.013
	Muslim	344	13.64				
	Protestant	479	14.69				
	Others§	85	14.98				
**Age**							
	17–19 years	420	14.15	6.37	(2, 1938)	0.002	0.007
	20–24 years	1438	14.93				
	25–45 years	83	16.1				
**Faculty**							
	Medical sciences	594	14.42	1.293	(6, 1969)	0.257	0.004
	Public health	429	14.93				
	Business and economics	149	15.32				
	Technology	97	14.51				
	Humanities and social sciences	417	15.04				
	Law	182	15.04				
	Education	108	15.38				

* equal variance not assumed (p-value for Levene's test=0 .03)

§ catholic, “waqofetta”, no religion

Stigma related to condom had a normal distribution with median 9 and scores ranging from 4–20. The independent-samples t-test analysis showed that the mean scores of condom related stigma were significantly higher among females (p< 0.001, Eta squared=0.009), those who had the habit of frequently attending church/ mosque (p= 0.029, Eta squared= 0.002), and those who had urban origin (p= 0.005, Eta squared= 0.004) compared to their counter parts.

Generally, all females and most males expressed their negative outlook to wards condom in the focus group discussions. A second year female student expressed her strong disapproval of carrying condom as:

If I see a female student carrying condom with her, just…it is like a priest carrying pistol with him instead of a cross. If I see my boy friend carrying condom, I suspect that he is looking for other girls.

A third year male student also added:

If you are seen with condom, you are caught-red-handed. If you have a girl friend, your relationship will be spoiled immediately.

Some male participants had the view that the possession of condom is not as such a seriously stigmatized act. For example, a fourth year male student had described his view as:

Although there are students who discriminate those who carry condom with them, there are also others who do not take it that much seriously except some light critics designed for making fun. People of urban origin are of this type. I have some friends who carry condom with them; I never feel bad about them. I know that sexual practice is much more rampant than is usually reported.

A third year male student confessed what he feels if seen by others carrying condom with him as:

To tell the truth, I and my friends do not have a negative impression for people carrying condom with them. Even sometimes, we purposely show condom to others as if it were accidentally exposed to get the respect of practicing sex.

The one-way between-groups ANOVA test showed that stigma related to condom did not have a statistically significant difference among students of different educational year level (p= 0.111), ethnicities (p= 0.138), and faculties (p= 0.728) (table 3).

**Table 3:** Comparison of the mean scores of stigma to condom among the different groups of Jimma University students using One- way between-groups ANOVA and Independent-samples t-test, May, 2009.

characteristics		Number	Mean	T/F	Df	p-value	Eta squared
**Gender**							
	Male	1604	9.5	−4.277	1966	<0.001	0.009
	Female	364	10.46				
**Church/mosque attending habit**							
	Yes	1713	9.76	2.185	1951	0.029	0.002
	No	240	9.18				
**Place of origin**							
	Urban	940	9.94	2.839	1950	0.005	0.004
	Rural	1012	9.44				
**Level of education**							
	Year-one	1075	9.85	2.004	(3, 1963)	0.111	0.003
	Year-two	313	9.31				
	Year-three	298	9.45				
	Year-four or above	287	9.73				
**Ethnicity**							
	Amhara	616	9.58	1.67	(5, 1930)	0.138	0.004
	Oromo	830	9.62				
	Tigre	57	8.82				
	Gurage	135	9.60				
	Wolita	51	10.24				
	Others*	247	10.16				
**Faculty**							
	Medical sciences	594	9.83	0.604	(6, 1969)	0.728	0.002
	Public health	429	9.49				
	Business and economics	149	9.81				
	Technology	97	9.23				
	Humanities and social sciences	417	9.68				
	Law	182	9.74				
	Education	108	9.83				

Others*= include Sidama, Keffa, Silte, Agew, Gamo, Somale, Shinasha, Yem, Dawro, Hamer, hadya

## Discussion

The purpose of the study was to assess the value University students give for virginity and boy/ girl friend, and the stigma they have for condom. It has not investigated factors that influence values and attitudes other than socio-demographic characteristics of the respondents. Many of the socio-demographic factors are known to be unlikely or less likely to be changed with interventions.

However, describing an attribute in terms of background characteristics of the study subjects is a necessary step for further understanding. This has been done using a large random sample and strong statistical techniques. Qualitative techniques were combined with focus group discussion to elucidate the perceptions underlying the values and attitudes.

Male gender, rural origin, and church/ mosque attending habit were found to be associated with higher virginity value. Tradition in many parts of Ethiopia and religious teachings discourage sexual intercourse before marriage. The majority of males participated in the focus groups stressed two main points concerning the virginity of their anticipated sexual partners: girls retain pleasant memories from their past relationships, and the possibility of continuing with previous relationships. According to Cox FD, it is very hard to predict what the effects of non-marital sexual relationships will have on marital sexuality (11). Concerning their own virginity, some males confessed that the strong sexual feeling makes them practice sex in contrary to their value. Following puberty the sexual desires of males is known to be strong to the extent that inaccessibility stops them (11,12). Males of rural origin believed that sexual practice weakens academic performance, and it is not acceptable from religious point of view. Non-marital sexual involvement is known to reduce intellectual, social, and other areas of involvement (11).

Some males during the focus group were in favor of sexual intercourse before marriage for they believed premarital sex is necessary to rule out sexual incompatibility and females want experienced boys. These findings show the typical myths related to sexual intercourse (13). There is sufficient evidence that most sexual in-competencies are psychological (11,12) and over all sexual satisfaction is determined by one's mental and emotional processes rather than the tactile maneuvers. It is not compatibility with sex that leads to love; it is the presence of love that makes sex compatible (11). The tendency of females to be less concerned for their virginity and the virginity of their anticipated partners indicates the liberalization of sex. The freedom to move up socio-economically, to choose a partner, and to leave unsatisfactory relationships is intertwined with increased non-marital sex, increased divorce, increased functional polygamy, and increased single-parent households (2–6,12). The variation in virginity value among the different ethnic and religious groups needs further exploration.

Male students with no church/ mosque attending habit, and urban origin had higher values for boy/ girl friend compared to their counter parts strengthening the evidence of sex liberalization. Gender had very small effect size in the quantitative findings and it was not manifested in the qualitative findings. The advantages of having a boy/ girl friend which were manifested during the focus group discussions were its being source of pride, its being source of confidence, support and respect. The disadvantages were wastage of time, psychological trauma when the relationship breaks; loss of freedom imposed by partner, and decreased social interactions. These are common problems of a relationship based on only sexual commitment rather than commitment to a total relationship (11).

Students below the age of 20 or below the third year educational level had lower value to boy/ girl friend. A study on other sexual behaviors in Jimma University had similar findings (8,13,14). The finding that Muslims had lower values to boy/girl friend than Christians needs further study. A previous study had shown that Muslims were more absolutist than hedonistic in their sexual values (14). Boy/girl friend value did not vary among the different faculties showing that it was a common value.

Female gender, church/ mosque attending habit, and urban origin had shown a slightly higher level of stigma related to condom compared to their counter parts. Social expectations and religious teachings that emphasize chastity which indirectly criticize condom possession and/or use could be responsible. All the focus group participants equated possessing condom with practicing sex whether they stigmatized it or not. For example, those participants who feel good when they possess condom feel so because they perceive that others would conclude they are practicing sex.

The slightly higher stigma related to condom among the urban students may be caused by a reporting bias in the direction of mass media teachings among the rural students. Sexual intercourse with out condom was 44% more likely among students of rural origin compared to urban students (8). In the focus group discussion, students of urban origin were less stigmatizing the possession of condom. The absence of difference in the level of stigma with educational year level, ethnicities, and faculties may show that the factors affecting the stigma are similar among these different groups.

To conclude, in contrast to many males, most females do not value virginity. Many students of both genders feel that a boy/ girl friend is very important in the campus life. These are indicators of the liberalization of sex. Yet, most of the respondents stigmatize condom indicating that making condom accessible to university students must be complemented with efforts to minimize the stigma related to it. We also recommend that sex educators targeting university students should focus on problems of liberalized, unprotected sex in relation to success in life.
